# Reverse remodeling of left atrium assessed by cardiovascular magnetic resonance feature tracking in hypertrophic obstructive cardiomyopathy after septal myectomy

**DOI:** 10.1186/s12968-023-00915-2

**Published:** 2023-02-13

**Authors:** Shujuan Yang, Xiuyu Chen, Kankan Zhao, Shiqin Yu, Wenhao Dong, Jiaxin Wang, Kai Yang, Qiulan Yang, Xuan Ma, Zhixiang Dong, Lele Liu, Yanyan Song, Minjie Lu, Shuiyun Wang, Shihua Zhao

**Affiliations:** 1 MR Center, Fuwai Hospital, Chinese Academy of Medical Sciences & Peking Union Medical College/National Center for Cardiovascular Diseases, Beilishi Rd 167, Xicheng District, Beijing, 100037 China; 2 Paul C. Lauterbur Research Center for Biomedical Imaging, Shenzhen Institutes of Advanced Technology, Chinese Academy of Sciences, SZ University Town, Shenzhen, 518055 China; 3 Department of Cardiac Surgery, Fuwai Hospital, Chinese Academy of Medical Sciences & Peking Union Medical College/National Center for Cardiovascular Diseases, Beilishi Rd 167, Xicheng District, Beijing, 100037 China

**Keywords:** Hypertrophic obstructive cardiomyopathy, Cardiovascular magnetic resonance, Left atrium, Strain, Surgery

## Abstract

**Background:**

Assessing the structure and function of left atrium (LA) is crucial in hypertrophic obstructive cardiomyopathy (HOCM) because LA remodeling correlates with atrial fibrillation. However, few studies have investigated the potential effect of myomectomy on LA phasic remodeling in HOCM after myectomy using cardiovascular magnetic resonance (CMR) feature tracking (FT). This study aims to evaluate the LA structural and functional remodeling with HOCM after myectomy by CMR-FT and to further investigate the determinants of LA reverse remodeling.

**Methods:**

In this single-center study, we retrospectively studied 88 patients with HOCM who received CMR before and after myectomy between January 2011 and June 2021. Preoperative and postoperative LA parameters derived from CMR-FT were compared, including LA reservoir function (total ejection fraction [EF], total strain [εs], peak positive strain rate [SRs]), conduit function (passive EF, passive strain [εe], peak early negative strain rate [SRe]) and booster function (booster EF, active strain [εa], late peak negative strain rate [SRa]). Eighty-six healthy participants were collected for comparison. Univariate and multivariate linear regression identified variables associated with the rate of change of εa.

**Results:**

Compared with preoperative parameters, LA reservoir function (total EF, εs, SRs), booster function (booster EF, εa, SRa), and SRe were significantly improved after myectomy (all *P* < 0.05), while no significant differences were observed in passive EF and εe. Postoperative patients with HOCM still had larger LA and worse LA function than healthy controls (all *P* < 0.05). After analyzing the rates of change in LA parameters, LA boost function, especially εa, showed the most dramatic improvement beyond the improvements in reservoir function, conduit function, and volume. In multivariable regression analysis, minimum LA volume index (adjusted β = − 0.39, *P* < 0.001) and Δleft ventricular outflow tract (LVOT) pressure gradient (adjusted β = − 0.29, *P* = 0.003) were significantly related to the rate of change of εa.

**Conclusions:**

Patients with HOCM after septal myectomy showed LA reverse remodeling with a reduction in LA size and restoration in LA reservoir and booster function but unchanged LA conduit function. Among volumetric and functional changes, booster function had the greatest improvement postoperatively. Besides, preoperative LAV_min_ index and ΔLVOT might be potential factors associated with the degree of improvement in εa.

**Supplementary Information:**

The online version contains supplementary material available at 10.1186/s12968-023-00915-2.

## Introduction

Left ventricular outflow tract (LVOT) obstruction is present in around three-quarters of patients with hypertrophic cardiomyopathy (HCM) [1]. Hypertrophic obstructive cardiomyopathy (HOCM), characterized by increased left ventricular (LV) filling pressures and progressive diastolic dysfunction, is one of the etiologies of heart failure with preserved ejection fraction. Left atrial (LA) enlargement and dysfunction are common in the clinical course of patients with HOCM. Given its crucial roles in predicting adverse cardiovascular events in HOCM [2, 3], LA structure and function are increasingly recognized as necessary indicators requiring close monitoring [4]. Previous studies demonstrated reduced LA dimension and volume in HOCM after surgery [5–8], but few have focused on LA phasic deformation function. Less affected by loading condition than volume, strain enables the quantification of the magnitude of myocardial deformation. As a sensitive tool, LA strain could be applied to evaluate the therapeutic effects [9], grade diastolic dysfunction [10], stratify the risk of atrial fibrillation (AF) [11, 12], and predict adverse cardiac events in HCM, even in patients with normal LA size and LV filling pressure [13].

Cardiovascular magnetic resonance (CMR) has been established as the gold standard imaging modality for assessing LA structure and function because of its high spatial resolution and superior tissue contrast in providing an accurate anatomic definition of thin asymmetric LA wall. Using routine cine images, CMR feature tracking (FT) is capable of quantifying LA volume and deformation parameters in different phases during the cardiac cycle. Nonetheless, data on the changes in FT-derived LA parameters after myectomy are sparse. The present study aims to evaluate the LA structural and functional remodeling in patients with HOCM after myectomy using CMR-FT and to further investigate the factors associated with the degree of LA reverse remodeling.

## Methods

### Study population

**Fig. 1:**
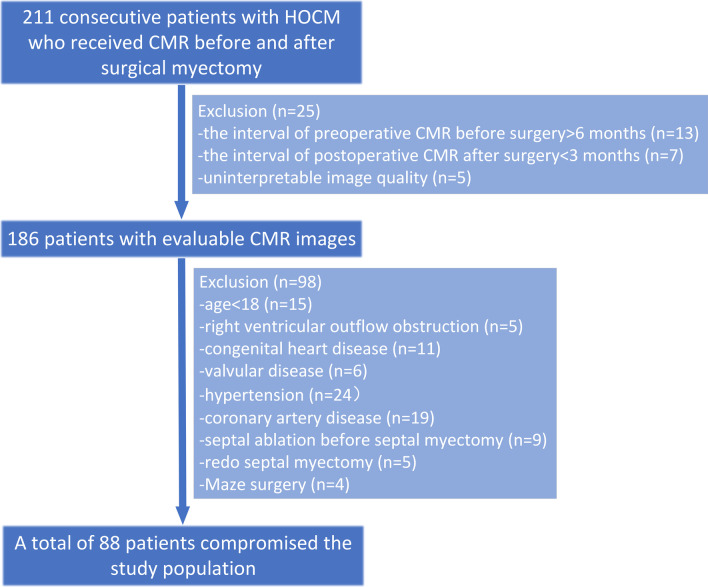
Flow chart of patient inclusion. *HOCM* hypertrophic obstructive cardiomyopathy, *CMR* cardiovascular magnetic resonance

The medical records of 211 consecutive patients with HOCM, who received CMR before and after surgical myectomy at Fuwai Hospital between January 2011 and June 2021, were retrospectively identified. All patients met diagnostic criteria and surgical indications for HOCM according to the guidelines [3]. The indications for surgical myectomy were mainly (1) severe symptoms, syncope or near-syncope despite optimal medical therapy, and (2) resting or provoked LVOT pressure gradient ≥ 50 mmHg. The detailed recruitment is shown in Fig. 1. The major exclusions were: (1) interval of preoperative CMR before surgery greater than 6 months and interval of postoperative CMR after surgery less than 3 months, (2) patients with uninterpretable image quality caused by the onset of arrhythmia including AF during CMR examination, (3) relevant comorbidities (e.g. congenital heart disease, valvular disease, coronary artery disease [coronary artery stenosis ≥ 30% at invasive coronary angiography or coronary computed tomography angiography], hypertension, infiltrative cardiomyopathy), (4) patients who underwent Maze surgery, septal ablation before myectomy, and redo myectomy. Finally, a total of 88 patients formed the study cohort. LVOT pressure gradient, mitral regurgitation (MR), and transmitral E/A ratio were assessed by echocardiography within 2 weeks before or after the pre- and postoperative CMR. According to Doppler echocardiographic criteria [14], including color flow MR jet, continuous wave signal of MR jet, vena contracta width, effective regurgitant orifice area, etc., MR was graded as 0 (none), 1 (mild), 2 (moderate), or 3 (severe). In addition, 86 healthy controls of similar age and sex without known cardiovascular or systemic disease were selected from our database for comparison [15]. The study conformed to the principles of the Helsinki Declaration, and the hospital institutional review board approved this study. Due to the retrospective design of this study, written informed consent was waived for patients with HOCM, and this study was granted permission by Fuwai Hospital to use clinical and imaging data of study population.

### CMR protocol

Images were acquired using 3 T CMR scanners (Ingenia, Philips Healthcare, Best, the Netherlands) with retrospective electrocardiographic (ECG) gating and 32-channel cardiac coil before and after myectomy. All patients (including 10 patients with a history of AF) were in sinus rhythm during pre- and postoperative image acquisition. Standard transverse and sagittal dark blood images were obtained using a semi-Fourier single-shot sequence with the following parameters: section thickness = 8 mm, section gap = 4 mm, matrix size = 224 × 192, field of view = 340 × 280 mm, repetition time = 2 heartbeats, echo time = 40 ms. Cine images (LV two- and four-chamber view and sequential short-axis planes covering the entire ventricles) were acquired using a balanced steady-state free precession sequence with typical parameters as follows: section thickness = 8 mm, section gap = 2 mm, matrix size = 224 × 256, field of view = 380 × 380 mm, repetition time = 2.8 ms, echo time = 1.4 ms, temporal resolution = 30–55 ms (depends on heart rate). The late gadolinium enhancement (LGE) images were obtained 10–15 min after intravenous administration of gadolinium-DTPA at a dose of 0.2 mmol/kg by using a segmented phase-sensitive inversion recovery Turbo FLASH sequence at the same position as cine images in end-diastole with the following parameters: matrix size = 256 × 162, field of view = 380 × 320 mm, slice thickness = 8 mm, slice gap = 2 mm, repetition time = 6.1 ms, echo time = 3 ms, flip angle = 25°, nominal inversion time = 300 ms.

### CMR analysis for left ventricle

All measurements were performed blind to the clinical and investigative data. To avoid measurement bias, the reader made postprocessing analysis in a random order rather than continuously processing pre- and postoperative study of the same patient. Quantitative LV measurements were obtained as described previously [16]. Briefly, using a postprocessing workstation (Intellispace portal, Philips), LV ejection fraction (LVEF), LV end-diastolic volume index, LV end-systolic volume index, stroke volume, cardiac index, and LV mass index were measured by drawing LV endocardial and epicardial contours (excluding papillary muscles) on a stack of LV short-axis cines. Maximum wall thickness and LGE percentage were measured offline with CVI42 (v. 5.1, Circle Cardiovascular Imaging, Calgary, Canada) (Additional file 1).

### CMR analysis for left atrium

**Fig. 2:**
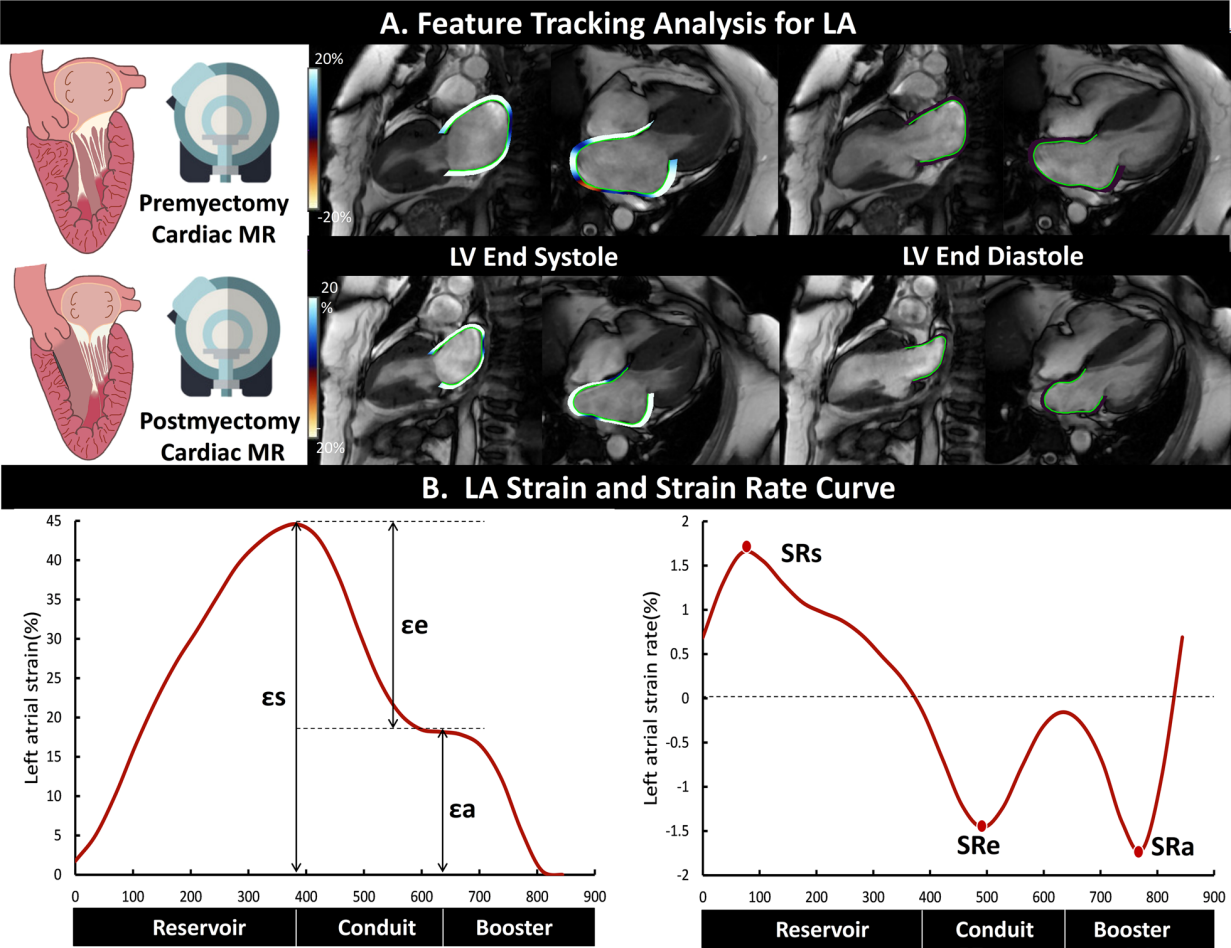
Example of measurement of left atrial (LA) phasic deformation function by feature tracking (**A**): the LA contours were semi-automatically delineated with manual adjustment at the phase of left ventricular (LV) end-systole and end-diastole on the two- and four-chamber cine images. Curves of feature tracking-derived LA strain and strain rate in different phases during the cardiac cycle in a healthy participant (**B**)

LA parameters were determined as previously described [17, 18]. LA anteroposterior and left–right diameters were measured on transverse dark blood images. Phasic volume, strain, and strain rate measurements of LA were obtained from FT (QStrain, Medis Suite 3.2, Leiden, the Netherlands). After determining the identical line connecting the mitral annulus and the most distal wall of the LA at its maximum and minimum volume on two- and four-chamber cines, LA endocardial borders were automatically delineated at LV end-systole and end-diastole (Fig. 2A). If necessary, manual adjustments were performed to exclude pulmonary veins and LA appendage and to attain optimal tracking. After being processed using the tracking algorithm, the contours were automatically propagated in all frames. Typical LA deformation parameter curves in a healthy subject are depicted in Fig. 2B. Three aspects of LA global strain were calculated: εs (total strain reflects LA reservoir function during LV systole), εe (passive strain reflects LA conduit function during early LV diastole), and εa (active strain reflects LA booster function during late LV diastole). Corresponding phasic strain rates were SRs (peak positive strain rate), SRe (peak early negative strain rate), and SRa (late peak negative strain rate), respectively.

Volumetric indices of phasic LA function were also obtained at LV end-systole (maximum LA volume [LAV_max_]), at LV diastole before LA contraction (passive LA volume [LAV_pac_]), and at the late LV end-diastole after LA contraction (minimum LA volume [LAV_min_]) [17, 18]. LA phasic emptying fractions (EF) were calculated as follows:

$${\text{LA}}\;{\text{total}}\;{\text{EF}} = \left[ {{{\left( {{\text{LAV}}_{\max } - {\text{LAV}}_{\min } } \right)} \mathord{\left/ {\vphantom {{\left( {{\text{LAV}}_{\max } - {\text{LAV}}_{\min } } \right)} {{\text{LAV}}_{\max } }}} \right. \kern-0pt} {{\text{LAV}}_{\max } }}} \right] \times 100\% ,$$

$${\text{LA}}\;{\text{passive}}\;{\text{EF}} = \left[ {{{\left( {{\text{LAV}}_{\max } - {\text{LAV}}_{{{\text{pac}}}} } \right)} \mathord{\left/ {\vphantom {{\left( {{\text{LAV}}_{\max } - {\text{LAV}}_{{{\text{pac}}}} } \right)} {{\text{LAV}}_{\max } }}} \right. \kern-0pt} {{\text{LAV}}_{\max } }}} \right] \times 100\% ,$$

$${\text{LA}}\;{\text{booster}}\;{\text{EF}} = \left[ {{{\left( {{\text{LAV}}_{{{\text{pac}}}} - {\text{LAV}}_{\min } } \right)} \mathord{\left/ {\vphantom {{\left( {{\text{LAV}}_{{{\text{pac}}}} - {\text{LAV}}_{\min } } \right)} {{\text{LAV}}_{{{\text{ac}}}} }}} \right. \kern-0pt} {{\text{LAV}}_{{{\text{ac}}}} }}} \right] \times 100\% .$$

### Cardiac surgery

All patients underwent an extended Morrow procedure performed by the same cardiac surgeon with 22 years of experience in cardiac surgery. As previously described [19, 20], a standard median sternotomy was performed following the cardiopulmonary bypass with ascending aortic cannulation and bicaval cannulation; Abnormal anatomic structures leading to LVOT obstruction, including hypertrophic septum myocardium and anomalous chordal attachments, were corrected. Elongated leaflets were not routinely folded unless severe MR or systolic anterior motion remained after resection. Mitral valve repair or replacement was performed if necessary. Fourteen patients received mitral valve repair (see Additional file 2: Table S1 for more detailed data on mitral valve repair), and no other concomitant surgeries were performed. The goal was to reduce the LVOT pressure gradient to less than 30 mmHg and to lower MR degree to none or mild, as confirmed by intraoperative transesophageal echocardiography.

### Statistical analysis

Statistical analyses were performed in SPSS (version 25.0, Statistical Package for the Social Sciences, International Business Machines, Inc., Armonk, New York, USA). Data were tested for normal distribution using the Shapiro–Wilk test, histograms, and Q–Q plots. Continuous data are expressed as mean ± standard deviations (SD) or as the median [interquartile range (IQR)] for variables with normal or non-normal distributions, respectively. Categorical data are presented as numbers and percentages. Parameters at baseline and follow-up CMR were compared using paired T-test or paired Wilcoxon signed-ranks test. Comparisons between different groups were performed using the independent samples *t*-test or Mann–Whitney U test for continuous variables, Chi‐square test or Fisher’s exact test for categorical variables. Associations of different continuous variables were examined by Pearson correlation coefficient or Spearman’s rank correlation coefficient (r). The delta value (Δ) equals the preoperative value minus the postoperative value. The rate of change is defined as the ratio of the delta value (Δ) to the preoperative value. Univariate and multivariate linear regression analyses (enter models) were used to find the potential variables associated with the rate of change of εa. Only those with a probability value < 0.05 by univariate analysis were entered in the multivariate model as covariates. Twenty subjects (including six healthy controls, seven premyectomy, and seven postmyectomy patients) were randomly selected from the entire cohort for the intra- and interobserver reproducibility assessment of LA parameters by the intra-class correlation coefficient (ICC) and Bland–Altman analyses. The intraobserver measurements were re-analyzed after 2 weeks. A two-sided *P* value < 0.05 was considered to indicate statistical significance.

## Results

### Participant characteristics

The baseline characteristics of 88 patients with HOCM are summarized in Table 1. The mean age at surgery was 44 ± 13 years old and about half were men (52%). The prominent symptom was chest tightness (71%). 86 healthy controls with similar demographic features were included for comparison (Table 1). Preoperative CMR was performed at a median of 14 days (IQR, 6 to 33 days) before myectomy. The interval between the pre- and postmyectomy CMR was at a median of 1.14 years (IQR, 1.01 to 1.56 years).

**Table 1:** Baseline characteristics of the study population

	HOCM patients (*n* = 88)	Healthy controls (*n* = 86)	*P* value
Age at surgery (years)	44.1 ± 13.1	44.0 ± 13.1	0.96
Sex			
Male	46 (52)	42(49)	0.65
Female	42 (48)	44(51)	0.65
Body surface area (m^2^)	1.8 ± 0.2	1.7 ± 0.2	0.31
Systolic blood pressure (mmHg)	114 ± 11	116 ± 11	0.25
Diastolic blood pressure (mmHg)	69 ± 9	71 ± 6	0.05
Smoker	22 (25)	–	–
Family history of HCM	19 (22)	–	–
Duration of HCM course (years)	6 (3, 10)	–	–
Diabetes	2 (3)	–	–
Hypercholesterolemia	17 (19)	–	–
New York Heart Association functional class			
I	1 (1)	–	–
II	25 (28)	–	–
III	60 (68)	–	–
IV	2 (2)	–	–
History of atrial fibrillation	10 (11)	–	–
Symptom			
Chest tightness	62 (71)	–	–
Dyspnea	42 (48)	–	–
Chest pain	26 (30)	–	–
Palpitation	19 (22)	–	–
Syncope	18 (21)	–	–
Medications		–	–
Beta-blockers	68 (77)	–	–
Calcium channel blockers	26 (30)	–	–
Diuretic	10 (11)	–	–
Antiarrhythmic	13 (15)	–	–
Echocardiography		–	–
Mitral regurgitation (none/mild/moderate/severe)	2/33/43/10	–	–
LV outflow tract pressure gradient (mmHg)	74 (60, 92)	–	–

Values are presented as n (%) and mean ± standard deviations or median (interquartile range)

*HOCM* hypertrophic obstructive cardiomyopathy, *LV* left ventricular

*P* value < 0.05 is considered to indicate statistical significance

### The effect of septal myectomy

#### Clinical benefits

**Fig. 3:**
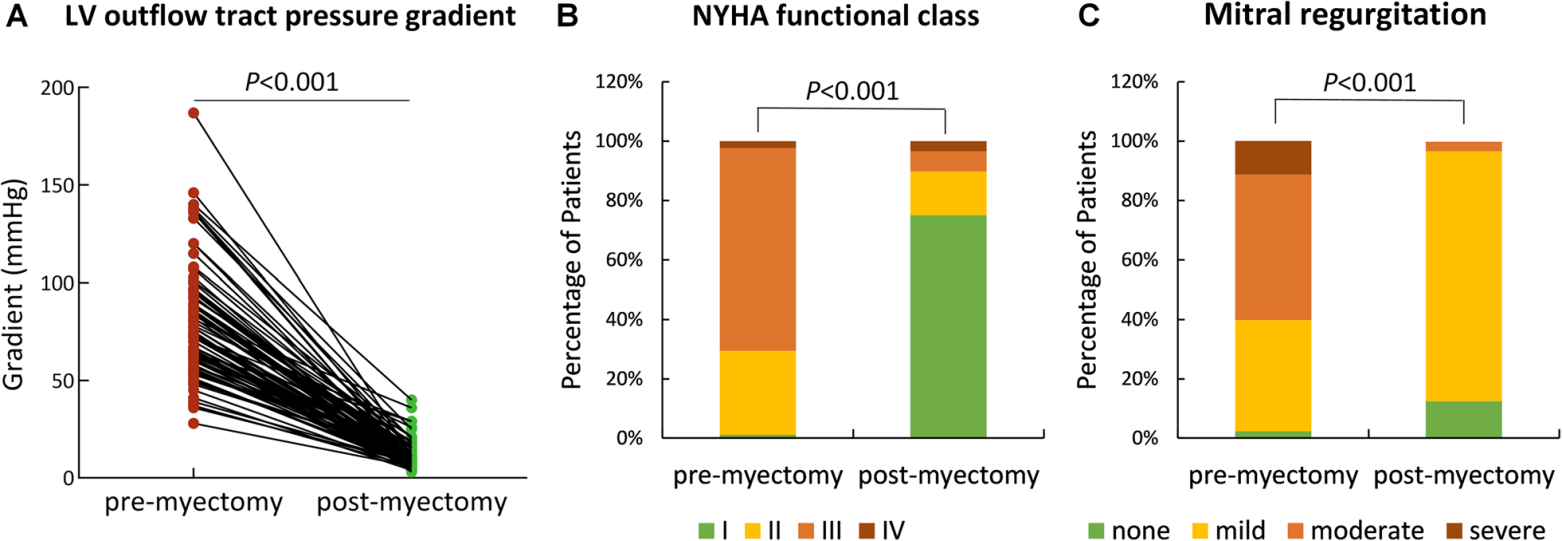
Comparisons of pre- and postmyectomy LV outflow tract pressure gradient (**A**), NYHA functional classes (**B**), and mitral regurgitation (**C**). *NYHA* New York Heart Association

As shown in Fig. 3, most patients experienced an improvement in symptoms after myectomy with 90% reporting NYHA class I or II functional capacity, yet only a proportion of 10% was reported before myectomy. The symptomatic benefit in patients with HOCM following myectomy was accompanied by a significant reduction in LVOT pressure gradient [74 (IQR, 60 to 92) mmHg vs. 9 (IQR, 7 to14) mmHg, *P* < 0.001]. The number of patients with none or mild MR before myectomy was 35 (40%), which increased to 85 (97%) postoperatively (*P* < 0.001).

#### Changes in LV conventional parameters

LV conventional parameters of study groups are displayed in Table 2. In patients with HOCM, LVEF, stroke volume, cardiac index, maximal LV wall thickness, and LV mass index were decreased postoperatively (all *P* < 0.001). Compared with controls, HOCM patients had higher LVEF, LV end-diastolic volume index, stroke volume, cardiac index, maximal LV wall thickness, and LV mass index before myectomy (all *P* < 0.05); Among these parameters, LV end-diastolic volume index, stroke volume, maximal LV wall thickness, and LV mass index remained elevated postoperatively, while LVEF was slightly lower than that of healthy controls (all *P* < 0.05).

**Table 2:** LV conventional parameters in control group and hypertrophic obstructive cardiomyopathy group before and after myectomy

Parameter	Premyectomy (n = 88)	Postmyectomy (n = 88)	Healthy controls (n = 86)	*P* value for premyectomy vs. postmyectomy
LV ejection fraction (%)	64.2 ± 8.1^†^	59.5 ± 7.6^†^	61.6 ± 4.7	< 0.001
LV end-diastolic volume index (ml/m^2^)	83.9 ± 24.4^†^	79.7 ± 23.3^†^	72.4 ± 13.0	0.12
LV end-systolic volume index (ml/m^2^)	27.8 (22.4, 35.6)	29.2 (24.7, 36.0)	28.2 (23.4, 32.4)	0.18
Stroke volume (ml)	92.9 ± 27.0^†^	82.9 ± 18.9^†^	77.2 ± 12.0	< 0.001
Cardiac index (l/min/m^2^)	3.46 ± 0.92^†^	3.07 ± 0.67	3.17 ± 0.64	< 0.001
Maximal LV wall thickness (mm)	28.5 ± 6.7^†^	23.1 ± 6.7^†^	9.6 ± 1.7	< 0.001
LV mass index (g/m^2^)	97.2 ± 50.9^†^	79.0 ± 41.2^†^	38.5 ± 9.3	< 0.001

Values are presented as mean ± standard deviations or median (interquartile range)

*LV* left ventricular

^†^Indicating *P* value < 0.05 for patients with hypertrophic obstructive cardiomyopathy versus healthy participants

#### Changes in LA structure and function

**Fig. 4:**
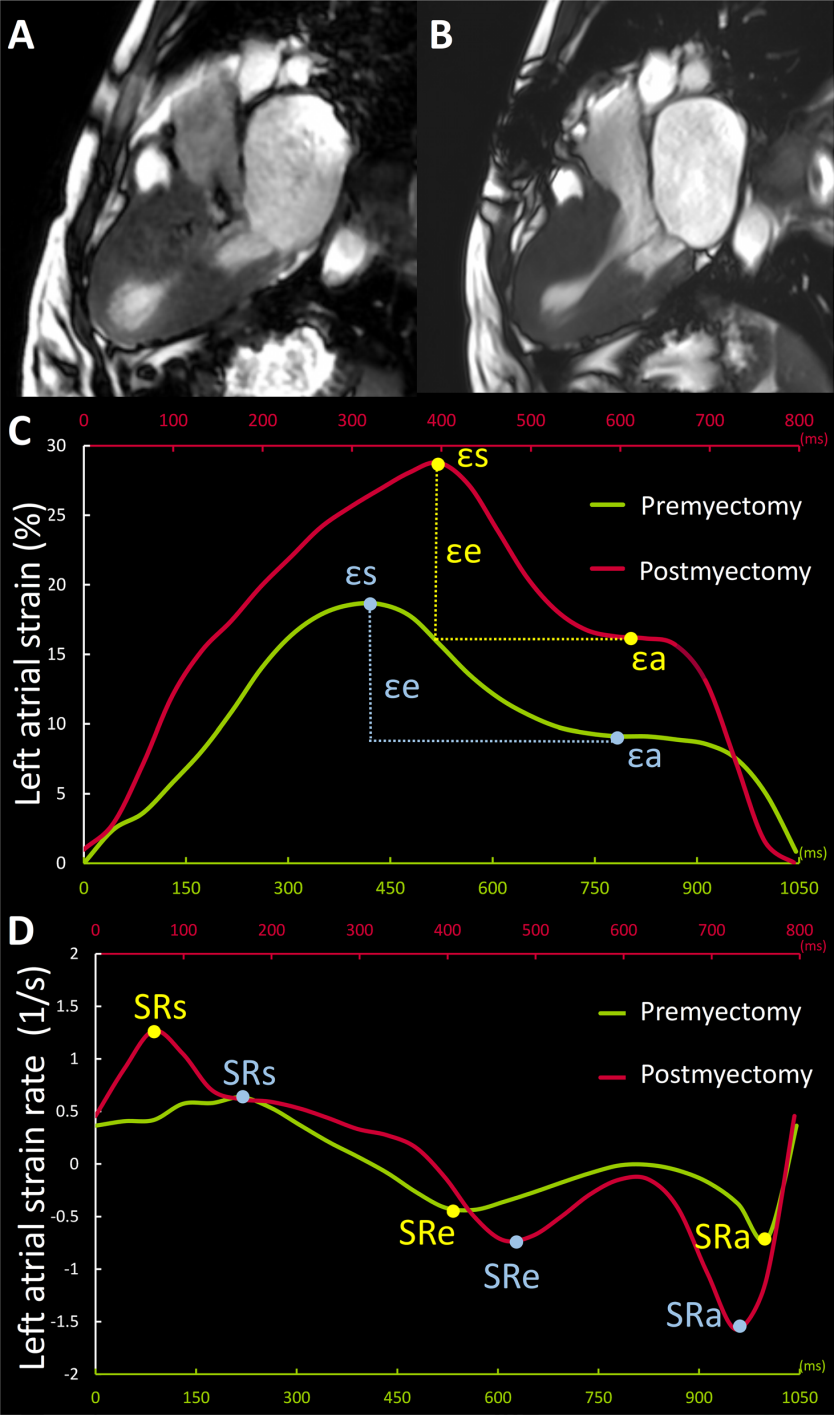
Representative case (52-year-old female) of a patient with hypertrophic obstructive cardiomyopathy who underwent septal myectomy. Upper row displays the LV outflow tract (LVOT) cine images at late systole. Systolic anterior motion (SAM) of mitral valve can be seen in preoperative cine (**A**). After surgery, LVOT is unobstructed without SAM (**B**). Lower rows display the curves of preoperative (in red) and postoperative (in green) LA strain (**C**) and strain rate (**D**) over the cardiac cycle

LA parameters of study groups assessed by CMR before and after myectomy are displayed in Table 3. LA anteroposterior diameter, LA left–right diameter, LAV_min_, LAV_pac_, and LAV_max_ were significantly decreased after myectomy (all *P* < 0.001). When comparing phasic functions, the LA reservoir function (LA total EF, εs, SRs) and booster function (LA booster EF, εa, SRa) were significantly improved postoperatively (all *P* < 0.05), but LA conduit function (LA passive EF, εe) was relatively preserved after myectomy, except for SRe [premyectomy, − 0.48 (IQR, − 0.32 to − 0.74) s^−1^ vs. postmyectomy, − 0.58 (IQR, − 0.41 to − 0.77) s^−1^, *P* = 0.007]. A representative case of LA phasic deformation function curves before and after myectomy is shown in Fig. 4*.* Although most LA indices improved after surgery, they were still worse than healthy controls (all *P* < 0.05).

**Table 3:** LA parameters in control group and hypertrophic obstructive cardiomyopathy group before and after myectomy

Parameter	Premyectomy (n = 88)	Postmyectomy (n = 88)	Healthy controls (n = 86)	*P* value for premyectomy vs. postmyectomy
LA structural parameter				
LA anteroposterior diameter (mm)	41 ± 8^†^	37 ± 8^†^	28 ± 6	< 0.001
LA left–right diameter (mm)	70 (63, 76)^†^	63 (58, 70)^†^	57 (51, 62)	< 0.001
LA V_max_ index (ml/m^2^)	69.3 ± 25.9^†^	50.0 ± 16.2^†^	36.9 ± 8.4	< 0.001
LA V_pac_ index (ml/m^2^)	48.4 (38.1, 71.2)^†^	38.3 (32.2, 44.9)^†^	23.4 (20.2, 26.9)	< 0.001
LA V_min_ index (ml/m^2^)	42.2 ± 24.4^†^	26.4 ± 15.8^†^	13.4 ± 4.1	< 0.001
LA reservoir function				
LA total EF (%)	42.5 ± 12.5^†^	49.4 ± 11.7^†^	64.0 ± 5.4	< 0.001
εs (%)	21.0 ± 9.8^†^	25.6 ± 9.5^†^	43.0 ± 9.0	< 0.001
SRs (1/s)	0.77 (0.53, 1.10)^†^	0.88 (0.67, 1.22)^†^	1.50 (1.33, 1.72)	0.003
LA conduit function				
LA passive EF (%)	18.5 ± 9.1^†^	19.6 ± 8.7^†^	33.8 ± 8.7	0.31
εe (%)	9.2 (5.8, 13.5)^†^	10.0 (7.0, 13.1)^†^	22.3 (17.9, 29.9)	0.21
SRe (1/s)	− 0.48 (− 0.32, − 0.74)^†^	− 0.58 (− 0.41, − 0.77)^†^	− 1.33 (− 1.11, − 1.75)	0.007
LA booster pump function				
LA booster EF (%)	29.7 ± 11.7^†^	37.5 ± 10.8^†^	45.4 ± 6.4	< 0.001
εa (%)	9.9 (6.0, 14.2)^†^	14.3 (11.0, 18.2)^†^	19.2 (16.6, 22.8)	< 0.001
SRa (1/s)	− 0.83 (− 0.54, − 1.26)^†^	− 1.17 (− 0.86, − 1.52)^†^	− 1.72 (− 1.47, − 2.10)	< 0.001

Values are presented as mean ± standard deviations or median (interquartile range)

*LA* left atrial, *EF* emptying fraction

^†^Indicating *P* value < 0.05 for patients with hypertrophic obstructive cardiomyopathy versus healthy participants

**Fig. 5:**
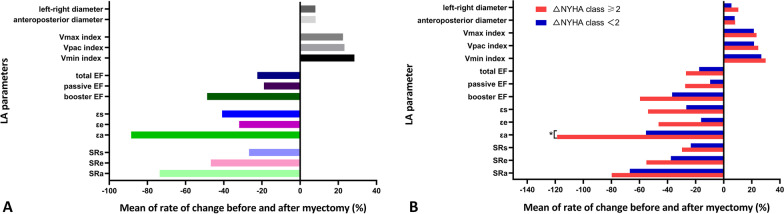
Bar graphs showing the rates of change of LA structural and functional parameters in all patients with HOCM after myectomy (**A**), and the comparisons for the rates of change of LA parameters between two subgroups defined by whether ΔNYHA function class was ≥ 2 after myectomy (**B**). *EF* ejection fraction, *LAV* LA volume. Delta value (Δ) = preoperative value − postoperative value, rate of change = delta value (Δ)/preoperative value × 100%

Furthermore, we investigated the rates of change in LA parameters, as they had different units and baseline levels, to assess the extent of improvement following myectomy (Fig. 5A). Overall, LA boost function, especially εa, showed the most dramatic improvement exceeding those seen in reservoir function, conduit function, and volume. After dividing patients into two subgroups according to whether a change in New York Heart Association (ΔNYHA) class was ≥ 2, the rate of change of εa in ΔNYHA class ≥ 2 group was significantly greater than the rate in ΔNYHA class < 2 group (*P* = 0.037). However, no statistical differences were observed when comparing other LA parameters (Fig. 5B).

### Associations between LA deformation parameters and LA conventional parameters

**Fig. 6:**
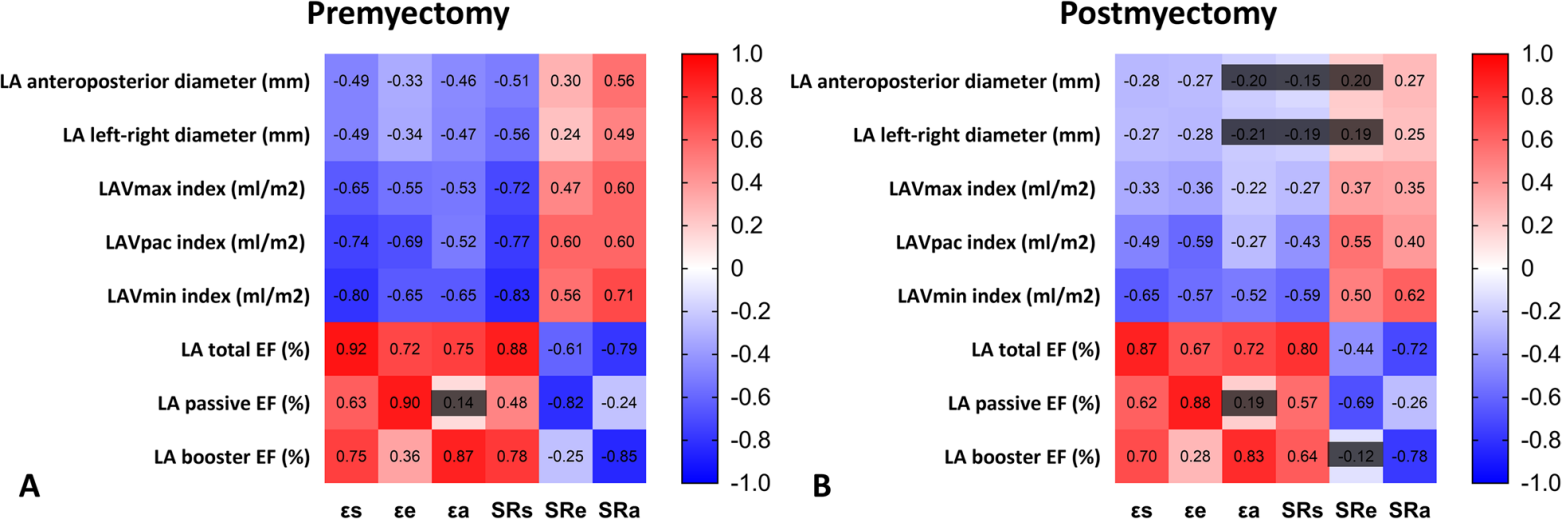
Preoperative (**A**) and postoperative (**B**) correlations of feature tracking-derived LA deformation parameters and LA conventional parameters. The number in each grid represents the correlation coefficient; Unshaded ones indicate that the correlations are statistically significant (*P* < 0.05)

The correlations between LA deformation parameters and LA conventional parameters are demonstrated in Fig. 6. Except for the correlation between preoperative εa and preoperative LA passive EF (*r* = 0.14, *P* = 0.46), almost all preoperative LA strains and strain rates were significantly correlated with preoperative LA conventional structural and functional parameters to various degrees (Fig. 6A). Nevertheless, postoperative correlations appeared to weaken, as most correlation coefficients were reduced after myectomy (Fig. 6B). Notably, the correlation between LA phasic deformation parameter and the corresponding LA phasic empty fraction was strongest both before and after myectomy.

### Factors associated with the rate of change of εa

Linear regression results are displayed in Table 4. Considering the collinearities among LA structural variables, we only included LAV_min_ index in linear regression analysis because of its highest correlation coefficient (r) with εa (from Fig. 6). In univariate analysis, systolic blood pressure, LAV_min_ index, ΔMR degree, and ΔLVOT pressure gradient were significantly associated with the rate of change of εa (all *P* < 0.05). In multivariate analysis, LAV_min_ index (adjusted *β* = − 0.39, *P* < 0.001) and ΔLVOT pressure gradient (adjusted *β* = − 0.29, *P* = 0.003) were significantly and independently related to the rate of change of εa.

**Table 4:** Univariate and multivariate analysis showing potential factors associated with the rate of change of εa

Variables	Univariate		Multivariate	
	*r*	*P*	Adjusted *β*	*P*
Age at surgery (year)	− 0.03	0.79		
Male	0.14	0.19		
Duration from myectomy to latest follow-up CMR (month)	0.05	0.68		
History of atrial fibrillation	− 0.10	0.37		
Preoperative beta-blockers	0.06	0.61		
Preoperative calcium channel blockers	− 0.11	0.31		
Postoperative beta-blockers	− 0.12	0.26		
Postoperative calcium channel blockers	0.07	0.54		
Systolic blood pressure (mmHg)	0.24	0.02	0.18	0.06
Diastolic blood pressure (mmHg)	0.06	0.58		
Mitral valve repair	− 0.14	0.20		
Mitral E/A ratio < 1	− 0.05	0.62		
Maximal LV wall thickness (mm)	0.13	0.22		
LV mass index (g/m^2^)	0.01	0.91		
Late gadolinium enhancement of LV (%)	0.08	0.46		
LAV_min_ index (ml/m^2^)	− 0.47	< 0.001	− 0.39	< 0.001
ΔMitral regurgitation degree	− 0.29	0.006	− 0.05	0.66
ΔLV outflow tract gradient (mmHg)	− 0.35	0.001	− 0.29	0.003
ΔStroke volume (ml)	− 0.19	0.082		

Delta value (Δ) = preoperative value − postoperative value, rate of change = delta value (Δ)/preoperative value × 100%

*LV* left ventricular, *LAV* left atrial volume

### Reproducibility of LA parameters derived from CMR-FT

**Fig. 7:**
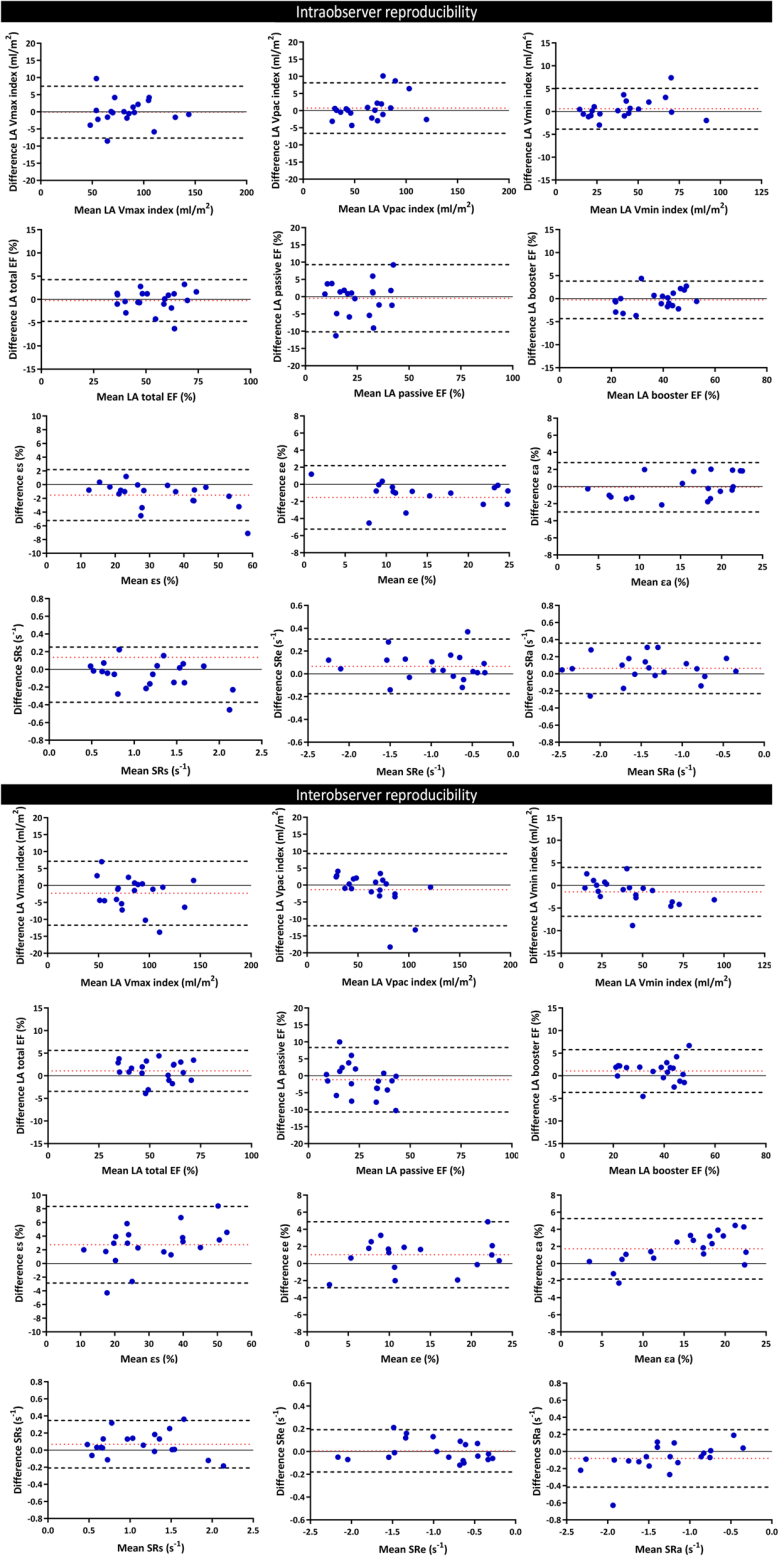
Bland–Altman plots illustrate intra- and inter-observer variability of the LA volumetric and deformation parameters derived from feature tracking. The bias (dotted red lines) and limits of agreement (dotted black lines) are shown in each graph

A detailed overview of the intra- and interobserver reproducibility of LA parameters derived from CMR-FT is displayed in Table 5 and Fig. 7. FT yielded excellent reliability (intraobserver ICC: 0.90 to 0.99; interobserver ICC: 0.88 to 0.99). Overall, Bland–Altman analyses showed better reproducibility for FT on the intraobserver level than the interobserver level, as evidenced by 95% limits of agreement. LAV_min_ index had the best reproducibility with the narrowest limits of agreement among LA volumetric parameters, and so did LA booster EF among LA emptying fractions.

**Table 5:** Intra- and interobserver reproducibility of the LA parameters derived from feature tracking

	Intraobserver reproducibility		Interobserver reproducibility	
	ICC	95% CI	ICC	95% CI
LAV_max_ (ml)	0.99	0.97–1.00	0.98	0.94–0.99
LAV_pac_ (ml)	0.99	0.97–1.00	0.99	0.98–1.00
LAV_min_ (ml)	0.99	0.99–1.00	0.99	0.98–1.00
LA total EF (%)	0.98	0.96–0.99	0.98	0.95–0.99
LA passive EF (%)	0.90	0.77–0.96	0.93	0.83–0.97
LA booster EF (%)	0.98	0.95–0.99	0.98	0.94–0.99
εs (%)	0.99	0.92–1.00	0.95	0.68–0.99
εe (%)	0.97	0.84–0.99	0.97	0.91–0.99
εa (%)	0.98	0.94–0.99	0.92	0.54–0.98
SRs (1/s)	0.90	0.74–0.96	0.88	0.73–0.95
SRe (1/s)	0.97	0.91–0.99	0.99	0.97–0.99
SRa (1/s)	0.97	0.91–0.99	0.95	0.85–0.98

*EF* emptying fraction, *LA* left atrial, *LAV* LA volume; *SR* strain rate

## Discussion

In this study, we performed FT analysis of 88 patients with HOCM using pre- and postmyectomy CMR. Our study demonstrated three main findings: (1) LA structure, reservoir function, and booster function partially recovered after the elimination of LVOT obstruction; (2) among all LA structural and functional parameters we investigated, εa showed the most dramatic improvement following myectomy, especially in patients with significant improvement in symptoms; (3) preoperative LAV_min_ index and ΔLVOT might be potential factors associated with the degree of improvement in εa after myectomy.

LA function in HOCM is gaining increasing importance as it is closely associated with the risk of AF occurrence, which exacerbates a symptomatic and functional decline and increases cardiac morbidity and mortality. LA function is complex and requires reproducible and precise cardiac imaging modality for assessment. Not only is CMR a superior imaging modality for evaluating LA, but LA phasic function assessed by CMR also provides prognostic value in HCM. In a recent study of 2755 patients with HCM, booster LA function measured by CMR was identified as a predictor of incident AF [12]. A smaller CMR study of 238 patients with HCM reported that reservoir and booster LA strain augmented the risk prediction of new-onset AF in HCM patients [11]. Considering the great improvement in reservoir and booster function after successful septal myectomy, especially in booster LA strain, we speculated that relieving LVOT obstruction would exert a positive effect on preventing the development of AF. Moreover, some echocardiographic studies proved that LA strain was predictive of heart failure events in HCM [10]. Our results also revealed that patients with significant symptomatic improvement had substantial improvement in LA active strain, suggesting a potential correlation between LA strain and symptoms in HOCM.

The concept of reverse LA remodeling after myectomy has been proposed both by CMR and echocardiographic studies. Similar to our results, Williams et al. [21] found elevated reservoir and booster function but unchanged conduit function after septal myectomy based on CMR-FT in 20 patients with HOCM. Amassing evidence showed close interaction between LA and LV function in three phases during each cardiac cycle [9]. LA reservoir function represents LA compliance, primarily influenced by the descent of the LV base driven by LV longitudinal shortening during systole [22]. Thus, the improvement in LV longitudinal strain in patients with HOCM after septal myectomy contributed to the increased LA reservoir function [23, 24]. In contrast to our results, an echocardiographic investigation reported a reduction in reservoir strain after myectomy [25]. Compared to this study, their cohort differed substantially in the percentages of patients with hypertension (36%), coronary heart disease (11%), and a history of AF (26%). Despite the positive effect of myectomy, these comorbidities would progressively deteriorate to impair LA function [11, 17, 26]. Therefore, it might be more indicative of the potential effect of myectomy on the LA restoration in our relatively homogeneous HCM cohort. Besides, there might be a systematic bias between different imaging modalities consequent to the discrepancy in results.

LA booster function reflects intrinsic LA contractility and LV end-diastolic pressure [9]. LA dilatation could be a physiological response to compensate for impaired LA booster function [17, 18]. Hence, the recovery of boost function was closely associated with reduced LA size accompanied by the decreased after-load. LA contractility also indirectly affected the movement of the atrioventricular ring toward the ventricles at the onset of LA relaxation [27], so we observed improvements in both reservoir and boost function.

LA conduit function relies on LV relaxation and LV wall stiffness [9]. Due to the persistence or even progression of myocardial fibrosis [20], which led to an irreversible increase in LV stiffness and further affected LV diastolic capacity [28, 29], LA conduit function did not improve significantly after the elimination of LVOT obstruction. Interestingly, we observed that a small number of patients still experienced new-onset AF after surgery, which might be related to the unimproved conduit function [30]. Larger studies with prognosis analysis are warranted to explore the associations between postoperative LA deformation function and new-onset AF after myectomy.

LA structure and function did not completely reverse in HOCM after surgery, which remained inferior to controls. This was possibly ascribed to irreversible LA fibrosis and underlying atrial myopathy. Playing a dominant role in LA structural, functional, and electrical remodeling [4], LA fibrosis is not only the atrial arrhythmia substrate [31, 32], but also impairs LA function [33]. However, accurate quantification of LA late gadolinium enhancement in routine practice remains challenging because of the technical limitation on the acquisition in the thin-walled atrium, confounders of peri-atrial fat and blood, and the time-consuming process. Measured by contrast-free cine sequences, LA deformation parameter could also reflect the severity of fibrosis burden [34, 35]. In terms of the excellent reproducibility and feasibility of CMR-FT for the assessment of LA deformation function shown in our study, it seems promising to monitor LA remodeling after surgery.

We also found that preoperative LAV_min_ index and ΔLVOT pressure gradient correlated with the rate of change of εa. LAV_min_ has been recently proposed as a better marker reflecting LV end-diastolic pressure, since the diminution of LA is a continuous and initiative process against the LV pressure during late diastole. A higher LAV_min_ index before myectomy means a more significant effect from elevated LV loading condition, so patients with higher preoperative LAV_min_ index could get more LA restoration after reducing afterload. Besides, evidenced by the nearly significant association between the degree of LA restoration with systolic blood pressure, our results indicated that LA function was susceptible to loading condition. The elevated LVOT pressure gradient is often accompanied by MR with varying severity, which is responsible for triggering the deterioration of LA strain. Our results suggested that LA contractile function of patients with a greater reduction in LVOT pressure gradient would benefit more from myectomy. Considering the potential for LA reversible functional remodeling based on favorable hemodynamic benefits and the susceptibility to loading condition of LA, our work underscored the importance of complete elimination of obstruction in patients with high LVOT pressure gradient.

### Limitations

Several limitations of this study must be recognized. First, there might be a selection bias as we excluded patients who hadn’t received either preoperative or postoperative CMR, primarily due to the long waiting time (average of 4 weeks) for CMR examination in our tertiary referral center. Second, our study population was limited, but to our knowledge, it was the largest cohort focusing on the changes in LA examined by CMR. Third, we did not make a prognosis analysis because of the small number of hard clinical events. Our patient inclusion time range was broad (approximately 10 years), and 47% of patients underwent myectomy after 2018, so their short-term outcomes were generally favorable. Forth, given the reported good–excellent inter-scan reproducibility of LA FT using long-axis cines [36–39], the inter-scan reproducibility was not tested in this study. Based on the relatively inferior scan-rescan reproducibility of LA phasic strain rates observed in previous study, our results on LA phasic strain rates should be interpreted with caution. Fifth, LA size was measured by bi-plane method, which is suboptimal to three-dimensional assessment from a short-axis stack covering LA. However, LA biplane assessment provides a reliable and applicable alternative to the time-consuming reference standard, and LA biplane parameters strongly correlate to those derived from Simpson’s methods [40]. Last, owing to the inherent drawbacks of retrospective study design, only transmitral E/A < 1 was available in this study among parameters of diastolic dysfunction assessed by echocardiography or cardiac catheterization. Prospective studies are needed to explore the correlation between diastolic function and LA deformation parameter.

## Conclusions

Our study demonstrated the feasibility of CMR-FT in assessing LA remodeling in patients with HOCM after septal myectomy. After successfully eliminating LVOT obstruction, they benefited from the relief of symptoms and partially recovered LA remodeling with a reduction in LA size and improvement in LA reservoir and booster function, whereas the LA conduit function seemed to be preserved. The improvement in LA boost function was beyond the improvement in reservoir function, conduit function, and atrial size following myectomy. Besides, preoperative LAV_min_ index and ΔLVOT might be potential factors associated with the degree of improvement in LA active strain. Future studies are needed to verify whether septal myectomy prevents the progression of AF in HOCM patients with postoperative LA reverse remodeling.

## Supplementary Information

**Additional file 1.** CMR postprocessing analysis for LV.

**Additional file 2.** Summary of mitral valve repair.

## Data Availability

The datasets used and analyzed during this study are available from the corresponding author upon reasonable request.

## Abbreviations

εa: Active strain
εe: Passive strain
εs: Total strain
AF: Atrial fibrillation
CMR: Cardiovascular magnetic resonance
ECG: Electrocardiogram
EF: Emptying fraction
FT: Feature tracking
HCM: Hypertrophic cardiomyopathy
HOCM: Hypertrophic obstructive cardiomyopathy
LA: Left atrial/atrium
LAV: LA volume
LAV_max_: Maximal left atrial volume
LAV_min_: Minimum left atrial volume
LAV_pac_: Passive LA volume
LGE: Late gadolinium enhancement
LV: Left ventricle/left ventricular
LVEF: Left ventricular ejection fraction
LVOT: LV outflow tract
MR: Mitral regurgitation.
NYHA: New York Heart Association
SRa: Late peak negative strain rate
SRe: Peak early negative strain rate
SRs: Peak positive strain rate
