# Supplementary material for: Reverse remodeling of left atrium assessed by cardiovascular magnetic resonance feature tracking in hypertrophic obstructive cardiomyopathy after septal myectomy
Source: J Cardiovasc Magn Reson. 2023 Feb 13;25:13. doi: 10.1186/s12968-023-00915-2 (PMC9923913; doi:10.1186/s12968-023-00915-2)
Supplement: Supplementary file 1 — Additional file 1. CMR postprocessing analysis for LV. [file 12968_2023_915_MOESM1_ESM.docx]

**METHODS**

**Cardiovascular magnetic resonance Analysis for Left Ventricle**

In the short 3D module of cvi^42^, LV endocardial and epicardial contours at end diastole were delineated. The myocardium in each slice of the short-axis cine stacks was then equally divided into 50 chords automatically at end-diastole, and maximum wall thickness was recorded by the maximum value of the corresponding chords. In the tissue characterization module of cvi^42^, endocardial and epicardial contours were traced without papillary muscles and trabeculations in short-axis late gadolinium enhancement stack. The extent of late gadolinium enhancement was quantified using the full width at the half-maximum method and expressed as a percentage of the left ventricular myocardial mass.
