# Supplementary material for: Reverse remodeling of left atrium assessed by cardiovascular magnetic resonance feature tracking in hypertrophic obstructive cardiomyopathy after septal myectomy
Source: J Cardiovasc Magn Reson. 2023 Feb 13;25:13. doi: 10.1186/s12968-023-00915-2 (PMC9923913; doi:10.1186/s12968-023-00915-2)
Supplement: Supplementary file 2 — Additional file 2. Summary of mitral valve repair. [file 12968_2023_915_MOESM2_ESM.docx]

**Additional table**

**Cardiac Surgery**

**Table S1. Summary of mitral valve repair in 88 patients with obstructive hypertrophic cardiomyopathy after myectomy**

| Variable | Number of patients |
| --- | --- |
| Mitral valve repair | 14 |
| Plication of anterior leaflet | 3 |
| Patch enlargement | 1 |
| Chordal or other attachment cutting | 6 |
| Papillary muscle resection | 7 |
| Chordal transposition | 1 |
| Edge to edge | 2 |
